# Construction of a four-mRNA prognostic signature with its ceRNA network in CESC

**DOI:** 10.1038/s41598-022-14732-7

**Published:** 2022-06-23

**Authors:** Lang Li, Qiusheng Guo, Gaochen Lan, Fei Liu, Wenwu Wang, Xianmei Lv

**Affiliations:** 1 Department of Hematology, Jinhua Hospital of Traditional Chinese Medicine, 439 West Shuangxi Road, Jinhua, 321017 China; 2 The Second Clinical Medical College, Zhejiang Chinese Medical University, 548 Binwen Road, Hangzhou, 310005 China; 3 Department of Oncology, The Second Affiliated Hospital of Fujian Medical University, 950 Donghai Street, Quanzhou, 362000 China; 4 Department of Dermatology, Jinhua People’s Hospital, 267 Danxi East Road, Jinhua, 321000 China; 5 Department of Oncology, The Third Affiliated People’s Hospital of Fujian University of Traditional Chinese Medicine, 363 Guobin Avenue, Fuzhou, 350108 China; 6 Department of Oncology, Quzhou Kecheng Hospital, 172 Shuanggang Road, Quzhou, 324000 China

**Keywords:** Cancer, Computational biology and bioinformatics, Biomarkers, Diseases

## Abstract

Cervical squamous cell carcinoma and endocervical adenocarcinoma (CESC) tumorigenesis involves a combination of multiple genetic alteration processes. Constructing a survival-associated competing endogenous RNA (ceRNA) network and a multi-mRNA-based prognostic signature model can help us better understand the complexity and genetic characteristics of CESC. In this study, the RNA-seq data and clinical information of CESC patients were downloaded from The Cancer Genome Atlas. Differentially expressed mRNAs, lncRNAs and miRNAs were identified with the edgeR R package. A four-mRNA prognostic signature was developed by multivariate Cox regression analysis. Kaplan–Meier survival with the log-rank tests was performed to assess survival rates. The relationships between overall survival (OS) and clinical parameters were evaluated by Cox regression analysis. A survival-associated ceRNA network was constructed with the multiMiR package and miRcode database. Kyoto encyclopedia of genes and genomes (KEGG) analysis and gene ontology analyses were used to identify the functional role of the ceRNA network in the prognosis of CESC. A total of 298 differentially expressed mRNAs, 8 miRNAs, and 29 lncRNAs were significantly associated with the prognosis of CESC. A prognostic signature model based on 4 mRNAs (OPN3, DAAM2, HENMT1, and CAVIN3) was developed, and the prognostic ability of this signature was indicated by the AUC of 0.726. Patients in the high-risk group exhibited significantly worse OS. The KEGG pathways, TGF-β and Cell adhesion molecules, were significantly enriched. In this study, a CESC-associated ceRNA network was constructed, and a multi-mRNA-based prognostic model for CESC was developed based on the ceRNA network, providing a new perspective for cancer pathogenesis research.

## Introduction

Cervical squamous cell carcinoma and endocervical adenocarcinoma (CESC) is one of the most common malignancies worldwide, with more than 570,000 new cases and 274,000 deaths per year^1,2^. The long-term survival rates of patients with early-stage disease have been greatly improved in recent years. However, the 5-year survival rate of patients with recurrent or metastatic disease remains less than 16.8%. Currently, the prediction of CESC prognosis mainly depends on the tumor-node-metastasis (TNM) stage. However, the TNM stage is based on anatomical information and does not reflect the biological heterogeneity of CESC. Hence, it is urgent to find novel biomarkers based on transcriptomics data that can act as prognostic indicators to guide precise individualized treatment.

Recently, the competing endogenous RNA (ceRNA) hypothesis has provided novel insights into the cancer research. CeRNA links the function of message RNA (mRNA) with long-noncoding RNA (lncRNA) and microRNA (miRNA)^3^. A ceRNA is a transcript targeted by a miRNA that sequesters the activity of the bound miRNA, effectively de-repressing other targets of that miRNA^4^. MiRNAs are small (20–22 nucleotides long) noncoding RNA that have been recognized as important negative regulators of mRNA translation^5^. LncRNAs are larger noncoding RNAs then miRNAs with more than 200 nucleotides^6^. The ceRNA hypothesis was proposed as a unique pathway for regulating the expression of RNAs^7^. The ceRNA hypothesis states that miRNAs act as the hub genes that suppress mRNA translation but lncRNAs compete for binding to one or more sites in miRNAs to suppress the function of miRNAs and participate in post-transcriptional control^8^.

Previous studies reported that the ceRNA networks might act as the biomarkers for prognosis in CESC. Song et al.^9^ constructed a CESC-associated ceRNA network which composed of 50 lncRNAs, 81 mRNAs and 18 miRNAs, and found that several RNAs were associated with the prognosis. Chen et al.^10^ constructed a CESC-associated ceRNA network composed of 17 lncRNAs, 5 miRNAs, and 7 mRNAs by weighted correlation network analysis (WGCNA), and found that E2F1 and hsa-mir-204 were related with worse prognosis. Ding et al.^11^ constructed a CESC-associated ceRNA network and revealed that *ADGRF4*, *ANXA8L1*, *HCAR3*, *IRF6* and *PDE2A* were associated with the prognosis. However, those studies did not construct a prognostic model based on the prognostic RNAs for CESC. CESC is a heterogeneous disease with multiple gene alterations and interactions. Hence, it is of great significance to construct a CESC-associated ceRNA network and develop a multigene prognostic model based on the ceRNA network.

**Figure 1:**
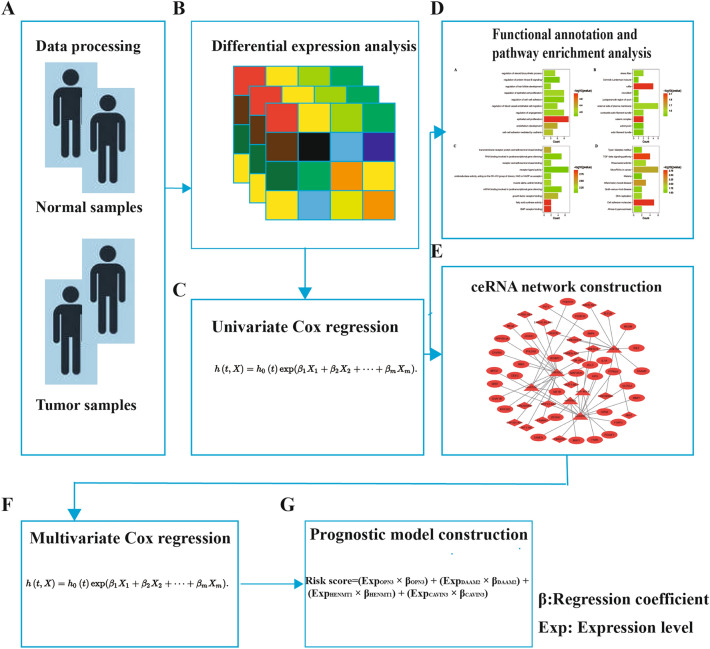
The workflow of the present study. (**A**) First, we downloaded the clinical information and RNA-seq data of CESC patients from The Cancer Genome Atlas (https://portal.gdc.cancer.gov) (TCGA) database. (**B**) Second, we performed differential expression analysis between normal and tumor samples. (**C**) Then, we used univariate Cox regression analysis to identify the prognosis-associated mRNAs, miRNAs, and lncRNAs. (**D**) Then, functional annotation and pathway enrichment were performed to investigate the identified prognosis-associated mRNAs. (**E**) In addition, we constructed a ceRNA network based on these prognosis-associated RNAs. (**F**) Next, multivariate Cox regression analysis was used to screen the prognostic mRNAs for development of the prognostic model construction. (**G**) Finally, the prognostic model was developed using the 4 mRNAs selected by screening.

As shown in the workflow diagram (Fig. 1), we first downloaded the clinical information and RNA-seq data of CESC patients from The Cancer Genome Atlas (https://portal.gdc.cancer.gov) (TCGA) database (Fig. 1A). Then, we performed differential expression analysis between normal and tumor samples (Fig. 1B). Then, we analyzed the relationships between differentially expressed RNAs and overall survival (OS) (Fig. 1C). Next, we utilized Kyoto encyclopedia of genes and genomes (KEGG) pathway enrichment analysis and gene ontology (GO) functional annotation to further investigate the function of the survival-associated RNAs (Fig. 1D). In addition, we constructed a ceRNA network based on those prognostic-associated RNAs (Fig. 1E). Afterward, four prognosis-associated mRNAs (*OPN3*, *DAAM2*, *HENMT1*, and *CAVIN3*) were identified through multivariate Cox regression (Fig. 1F). Finally, we developed a prognostic signature for CESC based on multiple mRNAs (Fig. 1G). This study aimed to provide a novel biomarker to guide personalized medicine and to facilitate an understanding of the molecular mechanisms for CESC.

## Results

### Differentially expressed RNAs analysis

**Figure 2:**
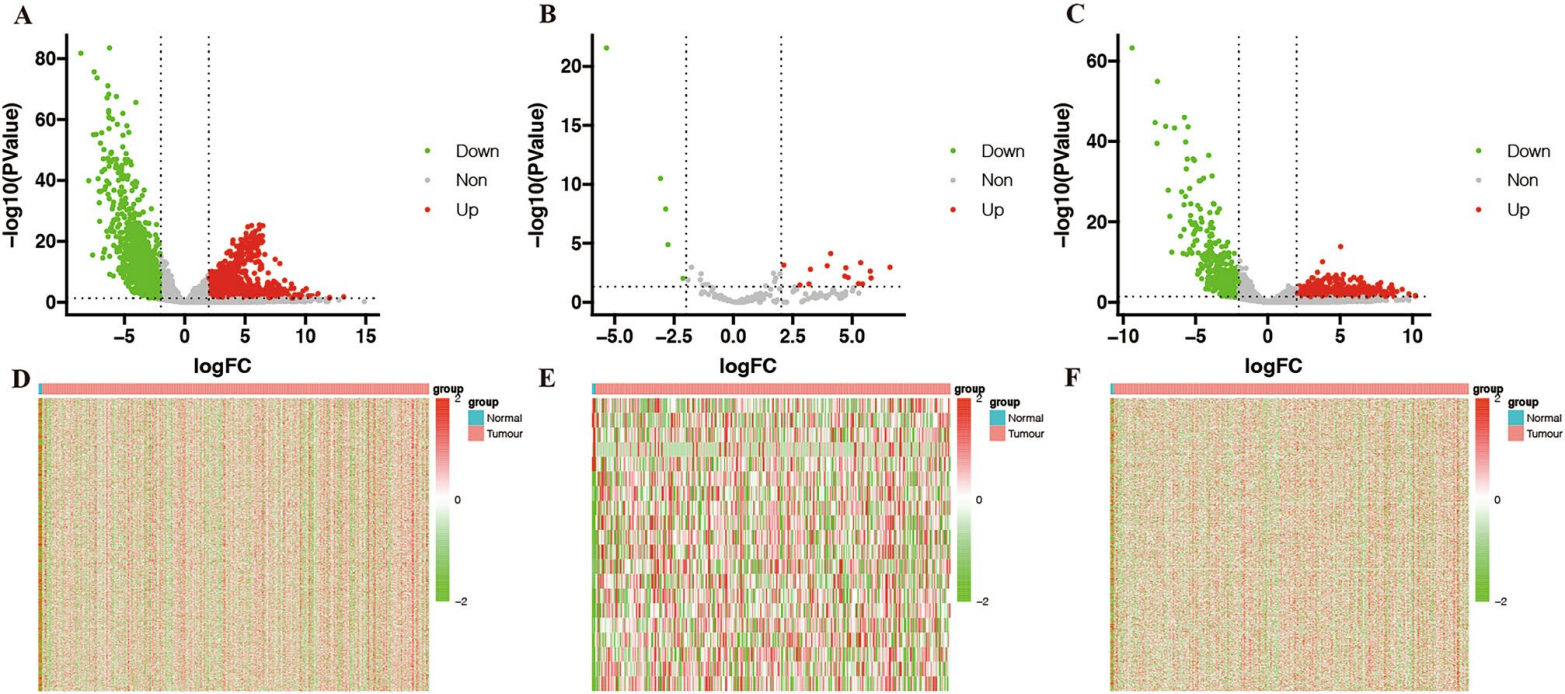
Differentially expressed RNAs between CESC and adjacent normal tissues. Volcano plot of the differentially expressed (**A**) mRNAs, (**B**) miRNAs and (**C**) lncRNAs. Red indicates high expression, and green indicates low expression. Black indicates no significant difference in expression. Heat map of the overall expression landscape of the differentially expressed (**D**) mRNAs, (**E**) miRNAs, and (**F**) lncRNAs.

In total, we identified 1398 downregulated and 1495 upregulated mRNAs (Fig. 2A), 5 downregulated and 15 upregulated miRNAs (Fig. 2B), and 493 downregulated and 658 upregulated lncRNAs (Fig. 2C), as shown in more detail in an additional file. A total of 19,545 mRNAs, 2713 miRNAs, and 13,977 lncRNAs were extracted from transcriptome data. The overall differential expression landscapes of mRNAs, miRNAs, and lncRNAs between 3 adjacent normal samples and 240 tumor samples are presented in Fig. 2D–F, respectively (see Additional file 1).

Please confirm the section headings are correctly identified.yes

### Survival-associated RNAs

**Figure 3:**
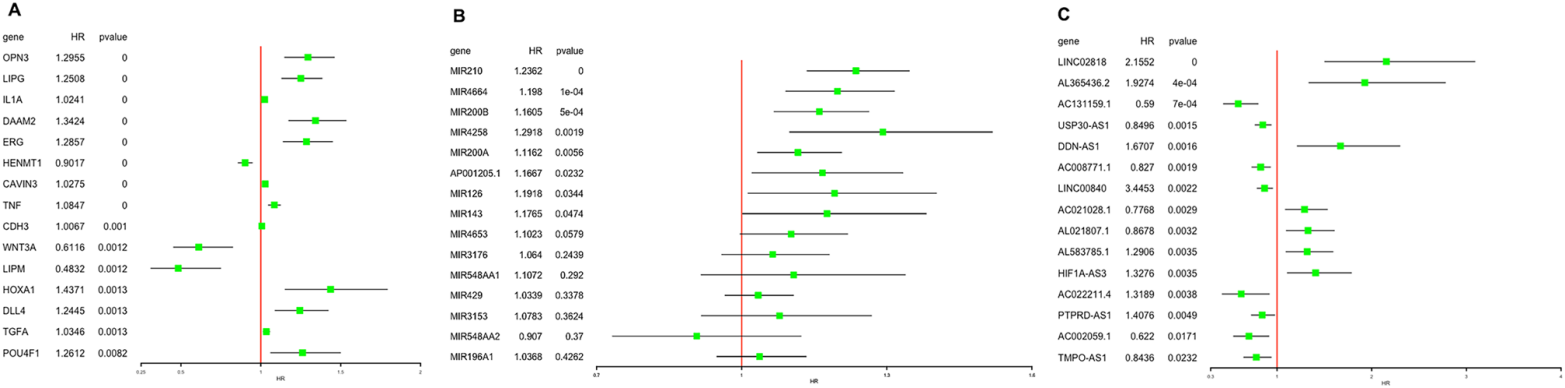
Forest plot of the hazard ratios of the top 15 survival-associated (**A**) mRNAs, (**B**) miRNAs, and (**C**) lncRNAs. A hazard ratio > 1 indicates the high-risk RNAs, and a hazard ratio < 1 indicates a protective RNAs.

The relationships between the differentially expressed lncRNAs, miRNAs, and mRNAs and OS were evaluated in 240 CESC patients. Univariate Cox regression analysis was used to identify overall survival related RNAs (prognostic RNAs). Finally, we found that 298 mRNAs, 8 miRNAs, and 129 lncRNAs were significantly associated with OS. The top 15 mRNAs, miRNAs, and lncRNAs ranked by p value are shown in Fig. 3A–C, respectively, as shown in more detail in an additional file (see Additional file 2).

### Functional annotation and pathway enrichment analysis

**Figure 4:**
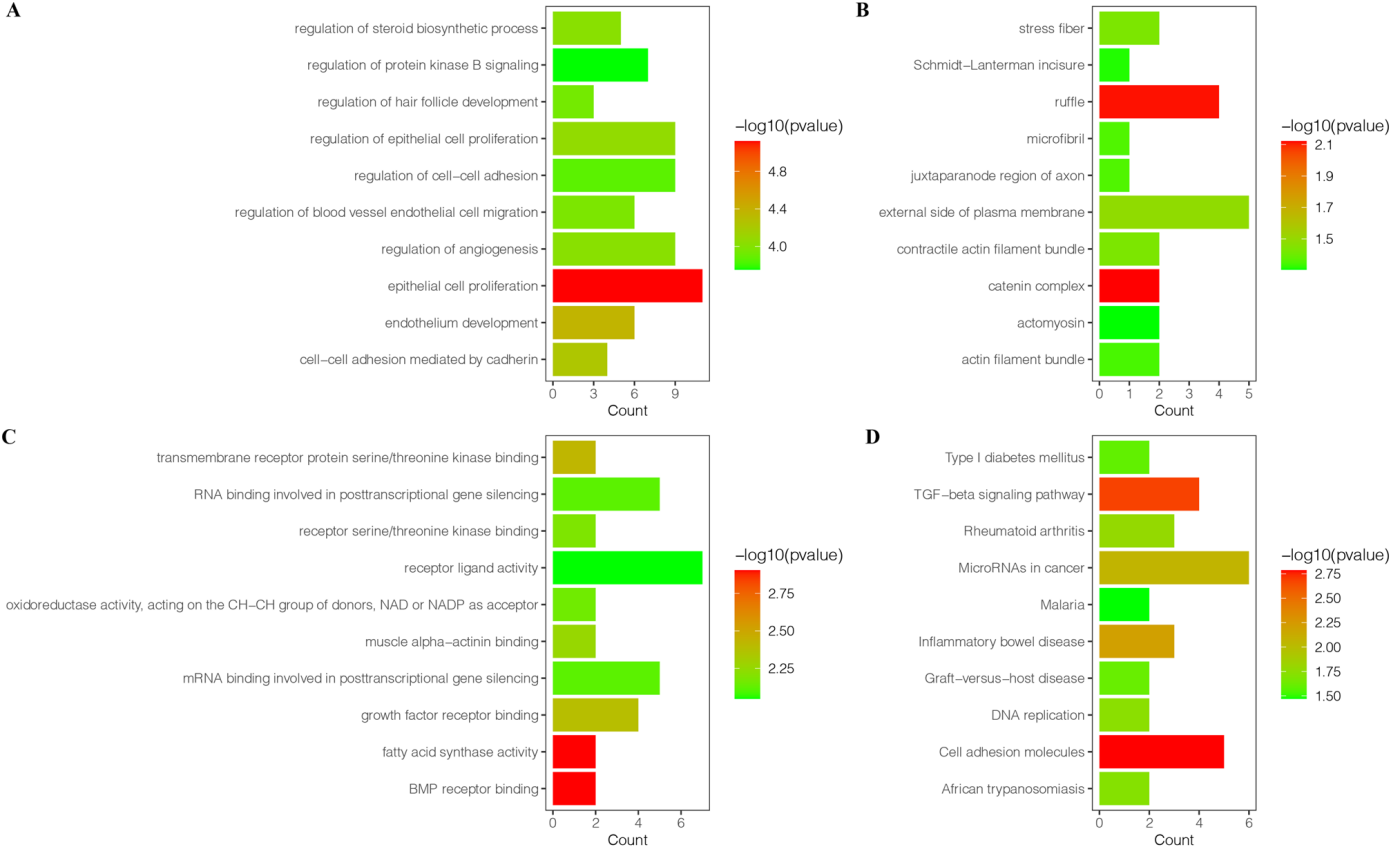
Enrichment analysis of the survival-associated RNAs. (**A**) Bar plot of enriched GO BP terms. (**B**) Bar plot of enriched GO CC terms. (**C**) Bar plot of enriched GO MF terms. (**D**) Bar plot of enriched KEGG pathways.

GO functional annotation and KEGG pathway enrichment analysis were utilized to investigate the biological functions of the prognostic mRNAs. GO functional annotations included biological process (BP), cellular component (CC), and molecular function (MF) annotations. The top 10 GO terms and KEGG pathways are listed in Fig. 4. GO BP analysis showed that the targeted mRNAs were significantly enriched in the terms epithelial cell proliferation and endothelial development (Fig. 4A). CC analysis revealed enrichment in the terms ruffle and catenin complex (Fig. 4B). In the MF analysis, the targeted mRNAs were significantly enriched in the terms BMP binding receptor and fatting acid synthase activity (Fig. 4C). KEGG pathway enrichment analysis revealed that the targeted genes were significantly enriched in the *TGF-β* and cell adhesion molecules signaling pathways (Fig. 4D). These results are shown in more detail in an additional file (see Additional file 3).

### Survival-associated ceRNA network

**Figure 5:**
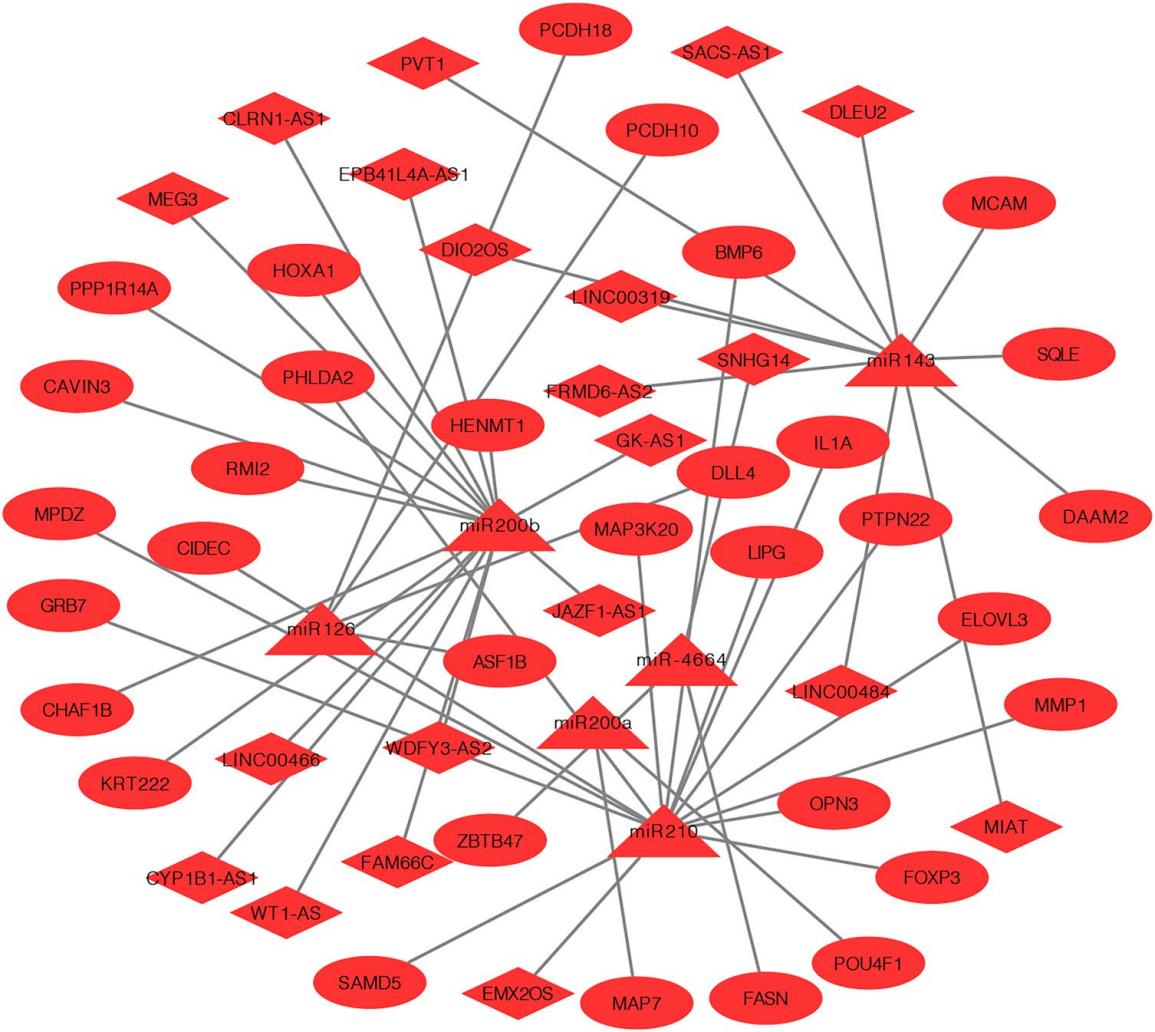
The prognostic ceRNA network in CESC. The connections indicate the interactions among lncRNAs, miRNAs, and mRNAs. The triangles represent miRNAs; the diamonds represent lncRNAs; the circles represent mRNAs.

A comprehensive survival-associated lncRNA-miRNA-mRNA ceRNA network was constructed by combining the lncRNA-miRNA interactions with the miRNA-mRNA interactions. The ceRNA network contained 24 lncRNAs, 6 miRNAs, and 34 mRNAs (Table S1, Fig. 5).

### Predictive model for overall survival

**Figure 6:**
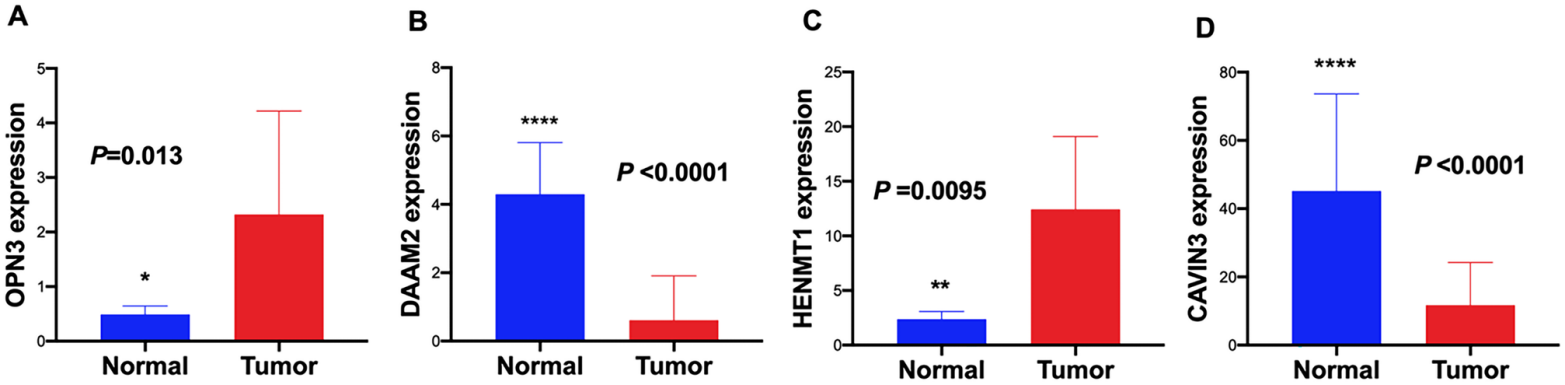
Comparison of the transcript levels of the 4 key mRNAs between CESC and normal tissues. The transcript levels of (**A**) *OPN3* and (**C**) *HENMT1* were significantly increased but the transcript levels of (**B**) *DAAM2* and (**D**) *CAVIN3* were significantly decreased in CESC tissues compared with normal tissues. *P < 0.05; **P < 0.01; ***P < 0.001; ****P < 0.0001 (unadjusted).

**Figure 7:**
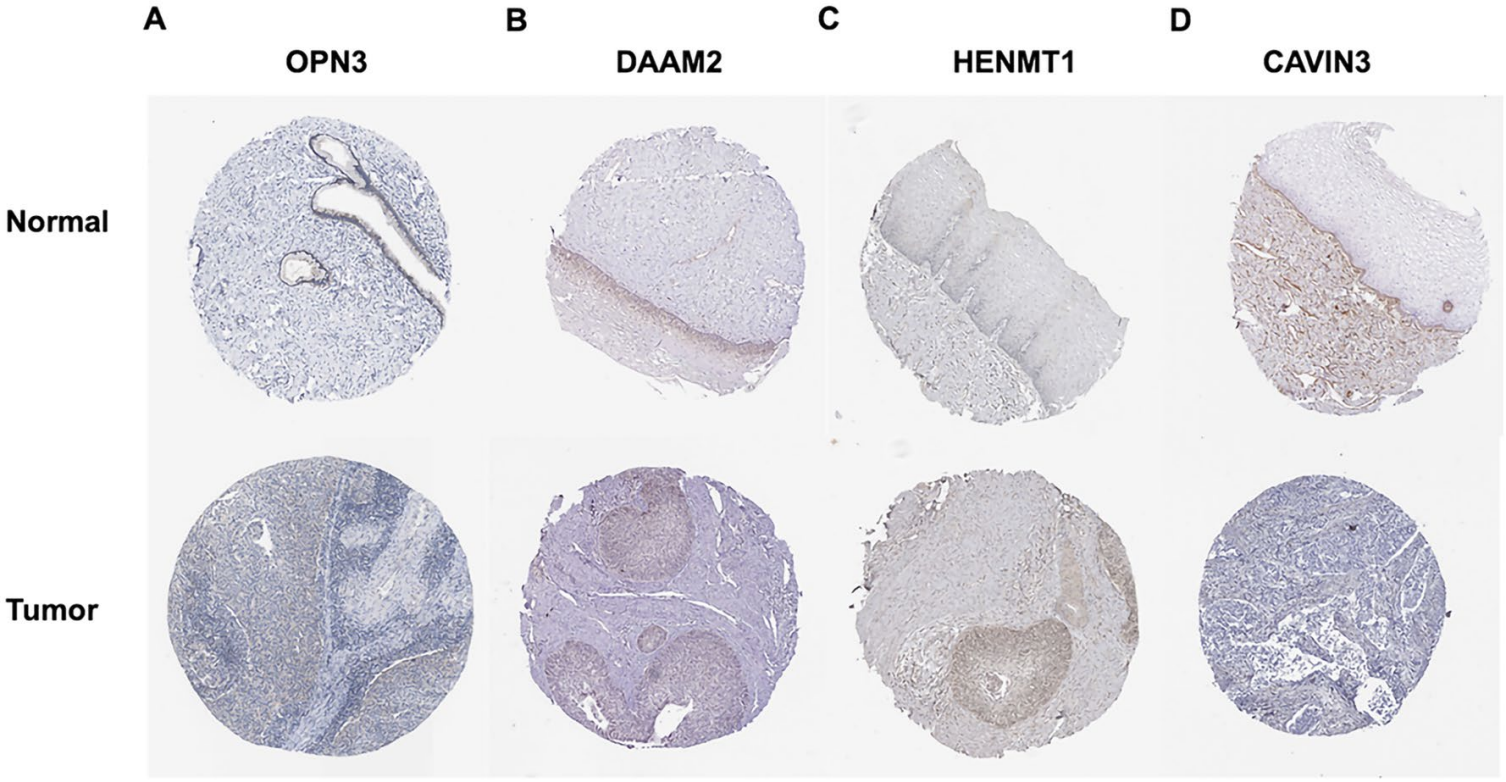
Representative immunohistochemical images of the 4 key mRNAs in CESC and adjacent normal tissues. The protein expression levels of OPN3 and HENMT1 were higher but the protein expression level of CAVIN3 was lower in CESC samples than those in normal samples (Human Protein Atlas).

To develop a multi-mRNA-based prognostic signature model for CESC, 8 mRNAs with *P* < 0.0001 (unadjusted) in the univariate Cox regression analysis were included in multivariate Cox regression analysis. Finally, 4 mRNAs (*OPN3*, *DAAM2*, *HENMT1*, and *CAVIN3)* were identified. Information on those mRNAs is shown in Table 1. In addition, we compared the transcript and protein levels of above 4 genes between adjacent normal and CESC tissues using a t test and immunohistochemistry (IHC). The transcript levels of *OPN3* (*P* = 0.013, unadjusted) and *HENMT1* (*P* = 0.0095, unadjusted) were significantly upregulated but the transcript levels of *DAAM2* (*P* < 0.0001, unadjusted) and *CAVIN3* (*P* < 0.0001, unadjusted) were significantly decreased in CESC tissues compared with adjacent normal tissues (Fig. 6). On the other hand, the Human Protein Atlas database was used to evaluate expression levels of the above 4 proteins in adjacent normal and CESC tissues as assessed by IHC. Based on the immunohistochemical staining images, the protein expression levels of *OPN3* and *HENMT1* were higher but the protein expression level of *CAVIN3* was lower in CESC samples than in normal samples, which was consistent with the transcriptomics data (Fig. 7).

**Table 1:** The information of 4 mRNAs significantly associated with OS in CESC.

Gene	Ensemble ID	Location	β	HR	*P*
OPN3	ENSG00000054277	Chr1(241,593,124–241,640,369)	0.2625	1.3002	0.0006
DAAM2	ENSG00000146122	Chr6 (397,923,66–399,048,70)	0.2724	1.3131	0.014
HENM1	ENSG00000162639	Chr1(108,648,295–108,661,474)	− 0.0794	0.9236	0.0046
CAVIN3	ENSG00000170955	Chr11(631,894,6–632,050,1)	0.0202	1.0204	0.0122

*β* regression coefficient, *HR* hazard ratio, *P P* value (unadjusted).

A multi-mRNA-based prognostic signature RS model was developed based on the above 4 mRNAs. The RS was calculated by the following equation: RS = (Exp_*OPN3*_ × β_*OPN3*_) + (Exp_*DAAM2*_ × β_*DAAM2*_) + (Exp_*HENMT1*_ × β_*HENMT1*_) + (Exp_*CAVIN3*_ × β_*CAVIN3*_). The “Exp” value represents the expression level and the “β” value represents the regression coefficient derived from the multivariate Cox regression model.

**Figure 8:**
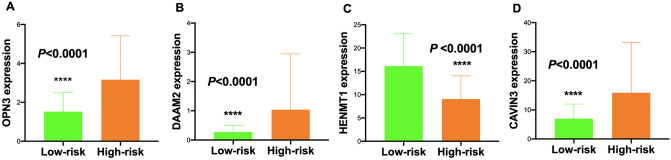
Comparison of the transcript levels of the 4 key mRNAs between the low-risk group and high-risk group. The expression levels of (**A**) OPN3, (**B**) DAAM2, and (**D**) CAVIN3 were higher but the expression level of (**C**) HENMT1 was lower in the high-risk group than those in the low-risk group. *P < 0.05; **P < 0.01; ***P < 0.001; ****P < 0.0001 (unadjusted).

**Figure 9:**
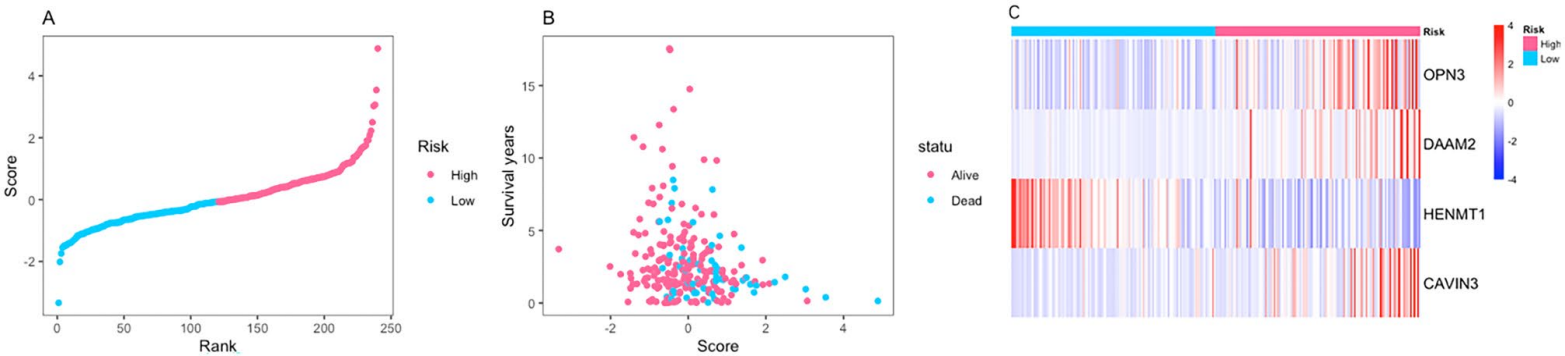
Performance of the prognostic model in classifying patients into the low-risk and high-risk groups. (**A**) Risk score distribution based on the four-mRNA signature. (**B**) Risk score distribution in the groups stratified by survival status based on the four-mRNA signature. (**C**) Heat map showing the expression of the 4 key mRNAs in low-risk and high-risk groups. The color from blue to red shows a trend from low expression to high expression.

The RS of each patient was calculated according to the above equation. Then, CESC patients were divided into a low-risk group and a high-risk group with the median RS as the cut-off value. A t test was used to compare the expression levels of the 4 mRNAs between the low-risk group and the high-risk group. The expression levels of *OPN3* (*P* < 0.0001, unadjusted), *DAAM2* (*P* < 0.0001, unadjusted), and *CAVIN3* (*P* < 0.0001, unadjusted) were higher but the expression levels of *HENMT1* (*P* < 0.0001, unadjusted) were lower in the high-risk group than those in the low-risk group (Fig. 8). Figure 9 shows the performance of the mRNA-based model. Figure 9A shows the ranking of patients according to the RS. The scatter plot shows that the OS of CESC patients decreased along with the increasing RS (Fig. 9B). The heat map shows that the expression level of *HENMT1* decreased but the expression levels of *OPN3*, *DAAM2* and *CAVIN3* increased with increasing RS (Fig. 9C).

**Figure 10:**
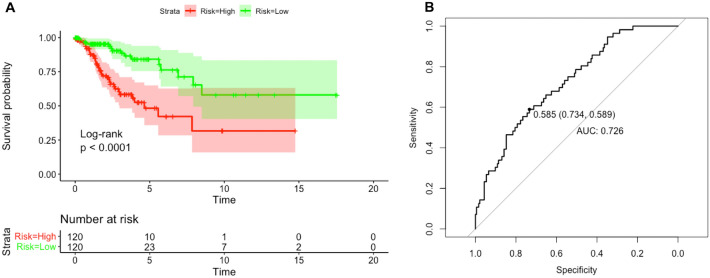
Kaplan–Meier and ROC curves based on the four-mRNA signature in CESC. (**A**) Kaplan–Meier survival curves showing OS outcomes according to relative high-risk and low-risk patients. (**B**) Time-dependent ROC analysis was performed to evaluate the prognostic ability of the four-mRNA signature for survival.

Kaplan–Meier survival analysis with the log-rank tests was performed to identify the relationships between different RS groups and OS, and the ROC curves were used to evaluate the sensitivity and specificity of the model. CESC patients in the high-risk group exhibited significantly shorter OS times (*P* < 0.0001, unadjusted) (Fig. 10A). The AUC of the RS (0.726) revealed that the model showed prognostic assessment ability (Fig. 10B).

Several clinical parameters were found to have some prognostic value by univariate analysis, for example, M stage (*P* = 0.02, unadjusted), N stage (*P* = 0.01, unadjusted), T stage (*P* = 0.001), pathological stage (*P* = 0.001, unadjusted) and the signature RS (*P* = 1.5e − 6, unadjusted); however, only the signature RS remained statistically significant with the confirmation by multivariate analysis (HR = 6.35, *P* = 0.01, unadjusted; Table 2).

**Table 2:** Univariate and multivariate Cox regression analysis of characteristics and Signature RS in CESC.

Clinical parameter	N	Univariate analysis		Multivariate analysis	
		HR (*95% CI*)	*P*	HR (*95% CI*)	*P*
**Age**		1.79(0.84–3.8)	0.13	–	–
≤ 65	192				
> 65	48				
**M stage**		3.55(1.2–10.55)	0.02	0 (0–lnf)	1
M0	107				
M1	10				
**N stage**		2.6(1.32–5.11)	0.01	1.51(0.56–4.06)	0.42
N0	125				
N1 +	53				
**T stage**		3.58(1.89–6.77)	0.001	3.58(1.89–6.77)	0.16
T1-T2	200				
T3-T4	26				
**Pathological stage**		5.77(3.01–11.05)	0.001	1.22(0.17–8.67)	0.84
I–II	125				
III–IV	53				
**Histological types**		1.13(0.55–2.31)	0.74	–	–
Adenosquam-ous	47				
Squamous	193				
**Signature RS**		0.22(0.12–0.41)	1.5e − 6	6.35(1.66–24.3)	0.01
High	120				
Low	120				

*N* number of patients, *HR* hazard ratio, *CI* confidence interval, *RS* risk score, *P P* value (unadjusted).

## Discussion

In this study, distinct mRNAs, lncRNAs, and miRNAs were identified to further understanding of the molecular events related to CESC prognosis. In addition, the constructed prognostic ceRNA network provided new insights into the prognosis of CESC.

CESC tumorigenesis involves a combination of multiple genetic alteration processes. However, almost all studies to date have focused on a single ‘driver’ gene or a single cluster of driver genes of CESC. Daniel et al.^12^ reported that Keratin-17 is a prognostic biomarker in CESC. Li et al.^13^ reported that *FAM83A* is a potential biomarker regulated by miR-20, which promotes the development of CESC through the PI3K/AKT/mTOR signaling pathway. To date, no single pivotal driver gene or gene cluster has been reported to be superior for evaluating the prognosis of CESC. Moreover, TNM staging, used as a major prognostic indicator, is based on anatomical information and does not reflect the biological heterogeneity of CESC. Hence, it is of great importance to construct a prognostic ceRNA network and develop a multi-mRNA-based model based on survival-associated biomarkers to predict the prognosis of CESC.

Through bioinformatics analysis, we found that 298 mRNAs, 8 miRNAs, and 129 lncRNAs were associated with the prognosis of CESC. In addition, we constructed a CESC-associated ceRNA network that contained 24 lncRNAs, 6 miRNAs, and 34 mRNAs from those prognostic RNAs. The ceRNA network can help us better understand the pathogenesis and prognosis of CESC from the multidimensional perspective of gene expression. We even developed a prognostic model using four key prognostic mRNAs (*OPN3*, *DAAM2*, *HENMT1*, and *CAVIN3*) in the ceRNA network, which showed excellent prognostic ability.

CeRNA networks of CESC have been constructed in other studies, but there are some limitations. Song et al.^9^ constructed a CESC-associated ceRNA network that consisted of 50 lncRNAs, 81 mRNAs and 18 miRNAs and found that several RNAs were associated with the prognosis. However, a prognostic model based on the prognostic RNAs for CESC was not developed in that study. In another study, Chen et al.^10^ also constructed a CESC-associated ceRNA network; however, the relationship between OS and only a single gene was assessed in that study. Although some researchers have constructed a CESC-associated ceRNA network and simultaneously developed a prognostic model, they did not use ROC curves to evaluate the prognostic ability of the model. Prognostic models based on multiple mRNAs could provide more accurate predictions than those based on single gene. We constructed a ceRNA network and identified several novel potential prognostic RNAs for CESC. Moreover, a prognostic model was developed based on the four key prognostic mRNAs. The prognostic ability of this model was indicated by AUC of 0.726 in ROC analysis.

Several prognostic models for CESC based on multiple genes have been developed in previous studies. Meng et al.^14^ developed a prognostic model for CESC based on *DSG2*, *ITM2A*, *CENPM*, *RIBC2*, and *MEIS2*. Liu et al.^15^ developed a multi-mRNA prognostic model composed of *ITGA5*, *HHEX*, and *S1PR4*. Similarly, we also developed a CESC-associated prognostic model based on *OPN3*, *DAAM2*, *HENMT1*, and *CAVIN3*, and the prognostic ability of this model was indicated by AUC of 0.726 in ROC analysis. Moreover, in our study, a Cox proportional hazards model was used, and we corrected for the following confounding factors that can greatly affect prognosis: T stage, N stage, M stage, and pathological stage. The RS signature of this signature was significantly associated with the clinical outcome of CESC patients. These results indicate that the signature we developed is an independent prognostic factor.

These four mRNAs have also been reported to play vital roles in tumorigenesis in various cancers. *OPN3* was found to be related to tumor metastasis and drug sensitivity. Chao et al.^16^ reported that *OPN3* enhanced tumor metastasis in lung adenocarcinoma. Jiao et al.^17^ revealed that *OPN3* sensitized hepatocellular carcinoma cells to 5-fluorouracil treatment by regulating the apoptotic pathway, a process was related to phospho-AKT and the Bcl2/Bax ratio. *DAAM2* drives tumorigenesis via various pathways. Chen et al.^18^ found that *DAAM2* promotes invasion in colorectal cancer by activating *PAK1* and promoting *MMP7* expression. Zhu et al.^19^ uncovered that *DAAM2* driven degradation of *VHL* promotes gliomagenesis. Consistent with our findings, Huang et al.^20^ reported that *HENMT1* plays protective roles in CESC. *CAVIN3* plays opposing roles in different types of cancers. Sun et al.^21^ found that *CAVIN3* promotes the migration, proliferation, and invasion of lung cancer cells and that process was related to the mammalian target of rapamycin (mTOR) signaling pathway. In contrast, *CAVIN3* functions as a metastasis suppressor by inhibiting the AKT pathway in breast cancer^22^. According to the above literature, we speculated that the AKT/mTOR signal pathway could be the primary enriched pathway of these four mRNAs.

In this study, we also discovered some prognostic miRNAs that extensively reported in previous studies. *MiRNA 210* was generally reported to exhibit oncogenic properties in breast, lung, head and neck, pancreatic cancer, and glioblastoma^23^. In consistent with our data, the overexpressed *miRNA 210* in breast cancer is related with a poor prognosis, that result is correlated with aggressiveness and shorter time to distant metastasis^24,25^. *MiRNA 200a* as a potential biomarker was widely reported to be associated with a poor prognosis in epithelial ovarian cancer^26^. *MiRNA 200b* was widely reported in cancer chemosensitivity, that process was related with EMT, cancer stem cells proliferation, angiogenesis, apoptosis, and cell cycle distribution^27^. *MiRNA 126* was reported to be a new and promising player in lung cancer that related to its promoting effect of metastasis and angiogenesis^28^. *MiRNA 4664*, *miRNA 4258*, and *AP001205.1* were rarely reported in previous studies and their oncogenic properties remained unknown, that would provide new lights on miRNA field. In summary, various studies indicated that our discovered prognostic miRNAs were potential biomarkers of cancers.

Excepted for *USP30-AS1* and *DDN-AS1*, few studies reported our discovered prognostic lncRNAs. Contrary to our data, *USP30-AS1* was reported to be related with poor prognosis in both primary and recurrent glioma patients^29^. *USP30-AS1* also promotes tumor cell survival by cis-regulating *USP30* and *ANKRD13A* in acute myeloid leukemia^30^. *DDN-AS1* was reported to be a poor prognosis factor with cervical cancer via DDN-AS1-miR-15a/16-TCF3 feedback loop regulates tumor progression^31^, that result support our study. All of these studies indicated that our discovered prognostic lncRNAs play vital roles in tumorigenesis and these unreported lncRNAs shed new lights on lncRNA filed.

The KEGG pathway analysis of the RNAs in the ceRNA network indicated that the targeted RNAs were significantly enriched in the *TGF-beta* signaling pathway and the cell adhesion molecules pathway. The TGF-beta pathway is a critical cancer-associated signaling pathway that is involved in proliferation, apoptosis, differentiation, migration, and epithelia-mesenchymal transition (EMT) of cancer^32,33^. The TGF-β signaling pathway also plays vital role in CESC. Deng et al.^34^ reported that *CD36* and *TGF-β* interact to promote the EMT in CESC. Yang et al.^35^ found that downregulation of *SEMA4C* inhibited EMT, invasion, and metastasis in CESC via inhibition of *TGF-β*. The cell adhesion molecules pathway plays a critical role in the development of CESC. Carvalho et al.^36^ reported that cell adhesion molecule L1 is associated with a poor prognosis. Biological process analysis revealed that the main biological process altered by the survival-associated RNAs in the ceRNA network is the epithelial cell proliferation. Cytokinetic homeostasis is controlled by the balance between cell proliferation and apoptosis. Previous studies have reported that excessive cell proliferation activity can lead to precancerous lesions in some types of cancer. Obara^37^ indicated that the epithelial cell proliferation activity is significantly increased in a stepwise manner from normal gallbladder mucosa to cancerous tissue. However, different pathways are usually perturbed by different molecules and thus need to be further investigated in the laboratory.

Our study still has some limitations. First, although the effects of *OPN3*, *DAAM2*, *HENTM1*, and *CAVIN3*, composing our prognostic signature, on tumorigenesis have been reported in other types of cancer^16,19,21,38^, their exact effects on CESC have yet to be fully elucidated and need to be verified by experiments. Second, the research data came from a single online database, and another independent cohort is needed to verify the above results in the future. Third, the information of cervical cancer patients obtained from TCGA should be assessed with another experimental method.

## Conclusion

In conclusion, we identified multiple potential prognostic markers and constructed a ceRNA network that provides novel insights for studying gene complexity in CESC and helps us better understand the pathogenesis and prognosis of CESC from the multidimensional perspective of gene expression. Here, we developed a multi-mRNA-based prognostic model that may compensate for limitations of the commonly used prognostic evaluation system. This study may provide novel biomarkers and facilitate the design of molecular targeted therapies for CESC. Although we investigated the potential prognostic value of these genes, due to work limitations, we did not perform an evaluation in another independent cohort to verify above results. In addition, the biological functions and mechanisms of above genes have yet to be fully elucidated. In summary, more experimental and clinical studies are needed to explore their functions and verify their prognostic value of these genes in the future.

## Materials and methods

### Data processing

The clinical information and RNA-seq data of CESC patients were downloaded from The Cancer Genome Atlas (https://portal.gdc.cancer.gov) (TCGA) database. The inclusion criteria were as follows: (1) Patients with complete clinical information, including T stage, M stage, N stage, pathological stage, survival status, age, and histological type; and (2) Patients with complete lncRNA-seq, miRNA-seq, and mRNA-seq data. Finally, 240 CESC samples and 3 adjacent non-tumor samples were examined. All of the human subjects/data were downloaded and analyzed for scientific purposes, which was in accordance with TCGA ethics approval.

### Differentially expressed RNAs analysis

The edgeR R package^39^ was used to identify differentially expressed mRNAs, lncRNAs, and miRNAs between CESC and adjacent non-tumor samples. A false discovery rate (FDR)-adjusted *P* value < 0.05 and an absolute log_2_ fold change |log_2_FC| value > 2 were considered to indicate statistical significance. The expression data for lncRNAs, miRNAs, and mRNAs were converted to log2(count + 1) values after normalization with the edgeR R package for further analysis.

### Survival analysis and development of the prognosis-associated signature

The survival R package was used for univariate Cox regression analysis to assess the relationships between differentially expressed RNAs (i.e., lncRNAs, miRNAs, and mRNAs) and OS, and mRNAs with a *P* < 0.05 (unadjusted) were considered to statistically significant and termed to prognostic RNAs^40^. To further develop a multi-mRNA-based prognostic model, prognostic mRNAs with a *P* < 0.001 (unadjusted) in the univariate Cox regression analysis were included in multivariate Cox regression analysis, and prognostic mRNAs with *P* < 0.05 (unadjusted) were considered to statistically significant. Finally, we obtained four mRNAs (*OPN3*, *DAAM2*, *HENTM1*, and *CAVIN3*) to for the development of the prognostic signature.

The mRNA-based prognostic signature risk score model was developed on the basis of a linear combination of the expression level (Exp) multiplied by the regression coefficient (β) derived from the multivariate Cox regression model and was represented by the following equation, as previously reported^41,42^: Risk Score (RS) = (Exp_*OPN3*_ × β_*OPN3*_) + (Exp_*DAAM2*_ × β_*DAAM2*_) + (Exp_*HENMT1*_ × β_*HENMT1*_) + (Exp_*CAVIN3*_ × β_*CAVIN3*_).

With the median RS as cut-off value, CESC patients were divided into a low-risk group and a high-risk group. Time-dependent receiver operating characteristic (ROC) analysis was applied to assess the prognostic accuracy of the model, and the area under the curve (AUC) was used to assess the specificity and sensitivity of the model. The pROC R package was used to perform the time-dependent ROC analysis^43^.

### Analysis of the protein expression levels of the 4 key mRNAs

The Human Protein Atlas (https://www.proteinatlas.org) provides tissue and cellular distribution information for approximately 26,000 human proteins. This information was obtained via immunoassay techniques (immunohistochemistry, immunofluorescence, and western blotting) to detect protein expression in 64 cancer cell lines, 48 normal human tissues and 20 tumor tissues^44^. In this study, we compared the expression levels of the 4 key genes (*OPN3*, *DAAM2*, *HENTM1*, and *CAVIN3*) between normal and CESC tissues using immunohistochemical images from the Human Protein Atlas database.

### Functional annotation and pathway enrichment analysis

The clusterProfiler R package^45^ and ggplot2 R package^46^ were used to process the data for prognostic mRNAs and visualize the enrichment results of gene ontology (GO) enrichment analysis and Kyoto encyclopedia of genes and genomes (KEGG) pathway enrichment analysis^47,48^. GO enrichment analysis included biological processes (BP), molecular functions (MF), and cellular components (CC). The statistical significance threshold was set at Benjamini–Hochberg (BH)-adjusted *P* value < 0.05 for enrichment analyses.

### ceRNA network construction

We constructed a ceRNA network based on the identified prognostic RNAs. The multiMiR R package^49^ and miRcode (http://www.mircode.org/) database were applied to predict miRNA-mRNA interactions and lncRNA-miRNA interactions. Finally, Cytoscape (version 3.7.0) software was utilized to visualize the ceRNA network based on the lncRNA-miRNA-mRNA axes by combining the lncRNA-miRNA interactions with the miRNA-target gene interactions^50^. In the ceRNA network, lncRNAs and mRNAs act as natural miRNA sponges to suppress miRNA functions by binding to one or more sites in miRNA.

### Statistical analysis

The relationships between different groups and the gene expression profiles were assessed by t tests, and *P* < 0.05 (unadjusted) was considered to statistical significance. Univariate Cox regression and multivariate Cox regression analysis was used to analyze the relationships between the clinicopathological parameters and OS. R software (version 4.0.2; https://mirrors.tuna.tsinghua.edu.cn/CRAN/) was used to generate figures and perform statistical analyses.Please note we have moved the section “Ethics approval and Patient consent for publication” to the end of the methods, as per house style.yes

### Ethics approval

All of the data involved to human was obtained reasonably basing on TCGA ethical statements.

### Patient consent for publication

Not applicable.

## Supplementary Information

Supplementary Information 1.

Supplementary Information 2.

Supplementary Information 3.

Supplementary Information 4.

## Data Availability

The datasets used during the current study are available from TCGA (https://portal.gdc.cancer.gov) and The Human Protein Atlas (https://www.proteinatlas.org). Analyzed data is available within supplementary information or from the corresponding author upon reasonable request.

## Abbreviations

CESC: Cervical squamous cell carcinoma and endocervical adenocarcinoma
TCGA: The Cancer Genome Atlas
ceRNA: Competitive endogenous RNA
KEGG: Kyoto encyclopedia of genes and genomes
GO: Gene ontology
BP: Biological progress
CC: Cellular components
MF: Molecular function
ROC: Receiver operating characteristic
AUC: Area under curve
|FC|: Absolute value of fold change
RNA-seq: RNA sequence
LncRNA: Long non-coding RNA
miRNA: MicroRNA
mRNA: Message RNA
EMT: Epithelia-mesenchymal transition
OS: Overall survival
RS: Risk score
HR: Hazard risk
Exp: Expression level
β: Regression coefficient
IHC: Immunohistochemistry
BMP: Bone morphogenetic protein
WGCNA: Weighted correlation network analysis
FDR: False discovery rate
