# Supplementary material for: Construction of a four-mRNA prognostic signature with its ceRNA network in CESC
Source: Sci Rep. 2022 Jun 23;12:10691. doi: 10.1038/s41598-022-14732-7 (PMC9226135; doi:10.1038/s41598-022-14732-7)
Supplement: Supplementary file 4 — Supplementary Information 4. [file 41598_2022_14732_MOESM4_ESM.pdf]

Construction of a four-mRNA prognostic signature with its ceRNA network in CESC

Lang Li [1 *], Qiusheng Guo [2*], Gaochen Lan [3*], Fei Liu [4], Wenwu Wang [5], Xianmei Lv [6✉]

[1] Department of Hematology, Jinhua Hospital of Traditional Chinese Medicine, 439 West Shuangxi Road, 321017 Jinhua, China; [2] The Second Clinical Medical College, Zhejiang Chinese Medical University, 548 Binwen Road, 310005 Hangzhou, China; [3] Department of Oncology, the Second Affiliated Hospital of Fujian Medical University, 950 Donghai Street, 362000 Quanzhou, China; [4] Department of Dermatology, Jinhua People's Hospital, 267 Danxi East Road, 321000 Jinhua, China; [5] Department of Oncology, the Third Affiliated People's Hospital of Fujian University of Traditional Chinese Medicine, 363 Guobin Avenue, 350108 Fuzhou, China; [6] Department of Oncology, Quzhou Kecheng Hospital, 172 Shuanggang Road, 324000 Quzhou, China.

✉Correspondence: Mrs. Xianmei LV, Department of Oncology, Quzhou Kecheng Hospital, 172 Shuanggang Road, 324000 Quzhou, China.

E-mail: 201911011511059@zcmu.edu.cn.

*These authors have contributed equally to this work and share first authorship.

Table S1. The information of RNAs in ceRNA network.

Types	Gene names
lncRNAs	COL18A1-AS1, SNHG14, EMX2OS, LINC00479, LINC00299, WDFY3-AS2, WT1-AS, MEG3, EPB41L4A-AS1, CYP1B1-AS1, CLRN1-AS1, GK-AS1, LINC00466, FAM66C, LINC00319, SACS-AS1, DLEU2, JAZF1-AS1, PVT1, DIO3OS, FRMD6-AS2, MIAT, LINC00484, CRNDE
miRNAs	miR-4664, miR-143, miR200a, miR-210, miR-126, miR-200b
mRNAs	ZBTB47, FASN, OPN3, LIPG, IL1A, MPDZ, FOXP3, BMP6, GRB7, PHLDA2, MAP3K20, CIDEC, MMP1, SAMD5, ELOVL3, HENMT1, PTPN22, CAVIN3, DAAM2, HOXA1, KRT222, CHAF1B, PPP1R14A, SQLE, CHAF1A, RMI2, MAP7, POU4F1, MCAM, DLL4, ASF1B, PCDH10, PCDH18, FBN1

lncRNA, long-noncoding RNA; miRNA, microRNA; mRNA, message RNA.
